# Case report: Bilateral central serous chorioretinopathy-like abnormalities in a man with pulmonary arterial hypertension

**DOI:** 10.3389/fmed.2022.983548

**Published:** 2022-08-01

**Authors:** Xinrong Zhou, Jingxiang Zhang, Limin Gu, Hao Zhou, Jingfa Zhang

**Affiliations:** ^1^Department of Ophthalmology, Shanghai General Hospital (Shanghai First People’s Hospital), Shanghai Jiao Tong University, Shanghai, China; ^2^National Clinical Research Center for Eye Diseases, Shanghai, China; ^3^Shanghai Key Laboratory of Ocular Fundus Diseases, Shanghai, China; ^4^Shanghai Engineering Center for Visual Science and Photomedicine, Shanghai, China; ^5^Shanghai Engineering Center for Precise Diagnosis and Treatment of Eye Diseases, Shanghai, China; ^6^Nursing Department, People’s Hospital of Huangdao District, Qingdao, China; ^7^Department of Ophthalmology, Shanghai Aier Eye Hospital, Shanghai, China

**Keywords:** pulmonary arterial hypertension, central serous chorioretinopathy, macular edema, case report, vascular disease, fundus abnormalities, hypertension - complications

## Abstract

**Background:**

Pulmonary arterial hypertension (PAH) leads to progressive increases in pulmonary vascular resistance, right heart failure, and death if left untreated. Ocular complications secondary to PAH were less reported. In this study, we reported a case of bilateral visual loss and metamorphopsia in a patient with PAH, who developed central serous chorioretinopathy (CSCR)-like abnormalities and optic disc atrophy.

**Case summary:**

A 45-year-old man presented with decreasing central vision and metamorphopsia in both eyes. He had a history of PAH and 6-year history of low-dose oral sildenafil treatment. Slit-lamp examination revealed prominent dilated and tortuous episcleral and conjunctival vessels. Ultrawide-field color picture showed retinal pigment epithelial mottling and atrophy in ring-like configurations. Ultrawide-field autofluorescence showed multiple irregular hyper-autofluorescence with a constellation-like pattern surrounding the optic nerve head and macular region. Optical coherence tomography angiography (OCTA) b-scan demonstrated CSCR-like changes. Swept-source optical coherence tomography (SS-OCT) analysis showed optic nerve atrophy with enlarged cup/disc ratio in right eye, which was confirmed with perimetry. Fluorescein angiography (FA) showed marked leakage of macula and optic nerve head with time, cystoid macular edema, early blocking with late staining of the flecks as shown in the backgrounds of infrared and autofluorescence, and mild leakage in peripheral retina. Indocyanine green angiography (ICGA) showed dilation, tortuosity and congestion of all vortex veins without obvious leakage.

**Conclusion:**

Undertreated PAH may cause the congestion of the choroid and induce CSCR-like abnormalities.

## Introduction

Pulmonary arterial hypertension (PAH), characterized as the occlusion of the pulmonary arterioles, leads to progressive increases in pulmonary vascular resistance, right heart failure, and death if left untreated (1, 2). PAH is owing to endothelial dysfunction and uncontrolled proliferation of pulmonary artery smooth muscle cells and fibroblasts (1). PAH is characterized by an imbalance of nitric oxide, prostacyclin and endothelin levels, and current therapies target these three pathways, such as sildenafil, ambrisentan, epoprostenol, etc. (3).

Sildenafil, phosphodiesterase type 5 inhibitor, is a potent vasodilator that has been used for erectile dysfunction and PAH (4). Sildenafil potentiates the smooth muscle relaxant properties of nitric oxide in vascular tissue through upregulation of intracellular levels of the second messengers cyclic guanosine monophosphate (cGMP) by inhibiting phosphodiesterase type 5-mediated degradation of cGMP and thus increase in nitric oxide (5). Phosphodiesterase type 5 is present in choroidal and retinal vessels, its inhibition by phosphodiesterase type 5 inhibitors might increase choroidal blood flow and cause vasodilation of the retinal and choroidal vasculature (5). It was reported that sildenafil appears to increase blood flow velocity significantly in the retrobulbar and choroidal circulation (6). Moreover, many studies suggested that sildenafil mediated an increase in choroidal blood flow, while with a lesser effect on the retinal vasculature (6). Due to weak cross-inhibition of phosphodiesterase type 6 in retina, especially photoreceptors, ocular side-effects were reported after taking sildenafil, including transient blurring of vision, photophobia, bluish haze in vision, serous macular detachment, central serous chorioretinopathy (CSCR), etc. (5, 7, 8), although the causal relationship remains to be elucidated.

A large randomized phase III trial, including 277 adults with PAH and treated with sildenafil, demonstrated that sildenafil dosing up to 80 mg three times daily was safe and well tolerated (9). Daily chronic dosing in this patient population was also safe in the eye, which was not associated with visual change and had no detrimental effect on visual function, such as best-corrected visual acuity (BCVA), contrast sensitivity, color vision, visual field, or intraocular pressure (9).

In this study, we described a case of bilateral visual loss and metamorphopsia in a patient with PAH, who was undertreated with long-term low-dose sildenafil, demonstrating CSCR-like abnormalities in both eyes and optic disc atrophy in right eye.

### Timeline

The present case showed that long-term undertreated PAH might cause the congestion of the choroid and induce CSCR-like abnormalities. The timeline for this patient was shown in Supplementary Figure 1.

## Case presentation

### Chief complaints

A 45-year-old man first noticed decreased central vision, blurred vision and metamorphopsia in both eyes in March 2021, which deteriorated within 1 month.

### History of present illness

He had a history of PAH, diagnosed in June 2015, with long-term use of low-dose sildenafil. He was alone without brothers or sisters, and his parents passed away about 10 years ago with no family history of PAH documented.

### History of past illness

His medical therapy for PAH included sildenafil (2.5 mg, bid) and ambrisentan (5 mg, qd). He was not previously treated with steroids.

### Physical examination

**FIGURE 1:**
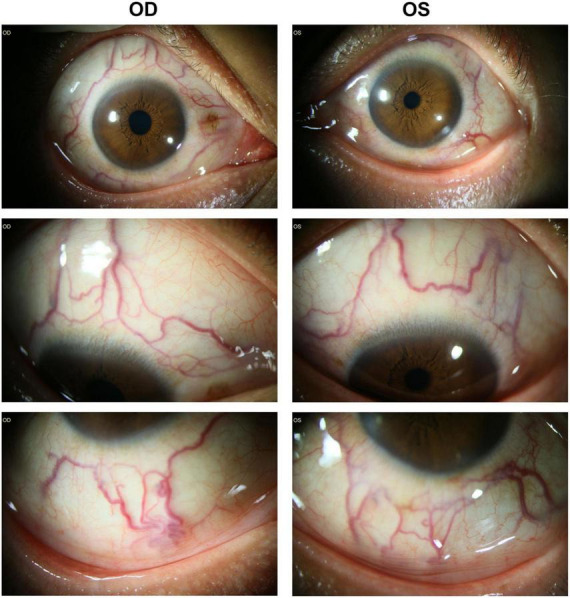
Prominent dilated episcleral and conjunctival vessels were noted in both eyes without signs of rubeosis of iris or iridocorneal angle closure. OD, right eye; OS, left eye.

The BCVA was finger count (FC)/10 cm in right eye and 0.82 (logMAR) in left eye. The mean refractive error was –3.25 diopter (right eye) and –1.00 diopter (left eye). His axial length was 23.49 mm (right eye) and 22.46 mm (left eye) detected by LENSTAR LS 900 (HAAG-STREIT, United States). The intraocular pressure was 19.9 mmHg (right eye) and 17.4 mmHg (left eye). Slit-lamp biomicroscope examination revealed prominent dilated and tortuous episcleral and conjunctival vessels in both eyes (Figure 1). Slit-lamp biomicroscopy of the anterior segment was normal without signs of rubeosis iridis, except for mild opacity of lens in both eyes. Ultrasonic biomicroscopy demonstrated normal anterior chamber depth, i.e., 2.39 mm (right eye) and 2.21 mm (left eye), and open anterior chamber angels (data not shown).

### Imaging examinations

**FIGURE 2:**
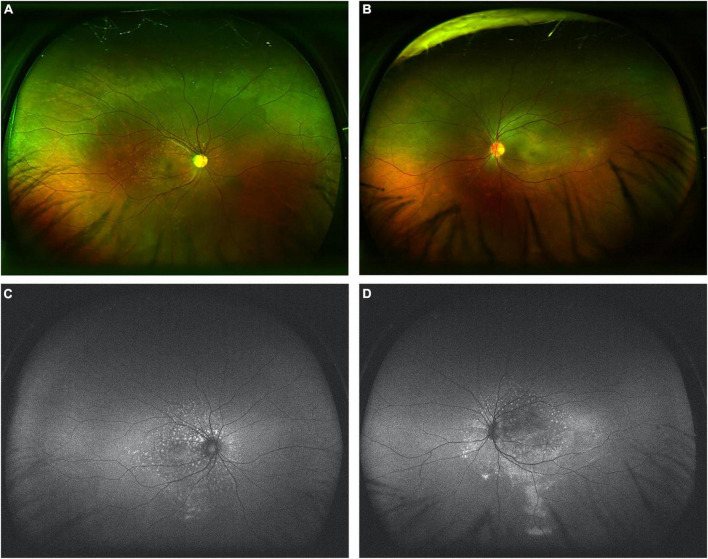
Ultrawide-field color pictures and autofluorescence in both eyes. **(A,B)** Ultrawide-field color pictures showed mild retinal venous dilation without hemorrhages and numerous flecks with subtle yellow subretinal flecks surrounding the papillomacular region. **(C,D)** Fundus autofluorescence showed mild hypo-autofluorescence in the foveal center and marked hyper-autofluorescence of these flecks surrounding the optic nerve head and macular region in both eyes. In left eye, gravitational track was observed on fundus autofluorescence.

In both eyes, flecked retinas were observed, and mild retinal venous dilation was noticed without hemorrhages. Ultrawide-field color picture (Optos 200Tx, United Kingdom) showed numerous flecks on retina in both eyes, especially in right eye, with subtle yellow subretinal flecks surrounding the macula and optic disc, as well as in the papillomacular region (Figures 2A,B). Retinal pigment epithelial mottling and atrophy in ring-like configurations in both eyes and optic nerve atrophy of right eye were also noticed (Figures 2A,B). Fundus autofluorescence using ultrawide-field imaging (Optos 200Tx, United Kingdom) showed mild hypo-autofluorescence in the foveal center and multiple irregular hyper-autofluorescent flecks with a constellation-like pattern surrounding the optic nerve head and macular region in both eyes (Figures 2C,D). In left eye, gravitational track was observed on fundus autofluorescence (Figure 2D).

**FIGURE 3:**
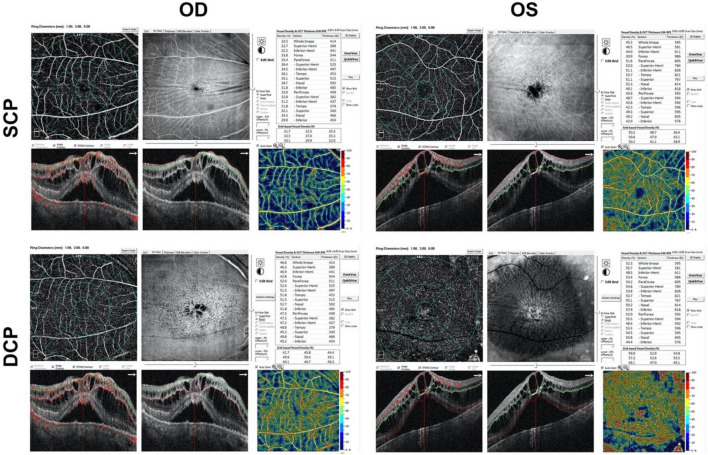
Marked cystoid macular edema with intraretinal fluid, subfoveal neurosensory retinal detachment and pachychoroid on OCTA b-scan in both eyes. OD, right eye; OS, left eye; SCP, superficial capillary plexus; DCP, deep capillary plexus.

Optical coherence tomography angiography (OCTA) (RTVue XR, Optovue Inc., Fremont, CA, United States) b-scan demonstrated chronic CSCR-like abnormalities in both eyes with both intraretinal and subretinal fluid accumulation, and choroidal thickening (Figure 3). The enface images (6 mm × 6 mm) of OCTA showed significant corrugation of the retina when segmented in superficial capillary plexus (SCP) and numerous cysts when segmented in deep capillary plexus (DCP) in both eyes. The thickness of central retina was higher in left eye than that in right eye, i.e., 595 μm vs. 414 μm (Figure 3), both demonstrating intraretinal cystoid edema and subretinal fluid, without obvious pigment epithelial detachment. The external limiting membrane was relative intact in right eye. There was shaggy photoreceptors and discontinuous ellipsoid zone in right eye. The vessel densities were also compared between both eyes, demonstrating less vessel densities in right eye than that in left eye in both SCP and DCP (Figure 3).

Since optic nerve atrophy of right eye was observed (Figure 2A), swept-source optical coherence tomography (SS-OCT) (DRI OCT Triton, TOPCON, Japan) analysis was performed on both optic discs and macula. The results showed obvious optic nerve atrophy with decreased thickness of the peripapillary retinal nerve fiber layer and enlarged cup/disc ratio in right eye, and mild inferior atrophy of the optic nerve in left eye (Supplementary Figure 2A). There was 32% symmetry for retinal nerve fiber layer thickness between right eye and left eye; and as for the thickness of retinal nerve fiber layer, SS-OCT analysis showed that significant decrease in the thickness of retinal nerve fiber layer in right eye than that in left eye, i.e., 74 μm vs. 166 μm (total), 73 μm vs. 159 μm (superior), and 76 μm vs. 142 μm (inferior) (Supplementary Figure 2A). Disc topography of SS-OCT showed the decreased rim area and disc area, while increased cup/disc ratio and cup volume in right eye compared with that in left eye (Supplementary Figure 2A). When comparison, the rim area was 0.12 mm^2^ (right eye) vs. 6.65 mm^2^ (left eye), and disc area was 1.59 mm^2^ (right eye) vs. 7.31 mm^2^ (left eye). The cup volume was also significantly increased in right eye, i.e., 0.44 mm^3^ (right eye) vs. 0.07 mm^3^ (left eye). Thus both retinal nerve fiber layer and disc topography analysis showed severe damage of the optic nerve in right eye than left eye (Supplementary Figure 2A). Consistent with the analysis on the optic disc with SS-OCT, the macular thickness analysis of inner retina was also demonstrated significantly thinner in right eye than that in left eye (Supplementary Figures 2B,C). SS-OCT analysis showed that the thicknesses were 61 μm (right eye) vs. 142 μm (left eye), 80 μm (right eye) vs. 115 μm (left eye), 142 μm (right eye) vs. 256 μm (left eye), respectively, for the total thickness of retinal nerve fiber layer, the total thickness of retinal nerve fiber layer and ganglion cell layer (+), and the total thickness of retinal nerve fiber layer, ganglion cell layer and inner plexiform layer (ganglion cell layer++) (Supplementary Figures 2B,C). To further confirm the above changes, the visual field was evaluated with a Humphrey Field Analyzer, model 750i (Zeiss Humphrey Systems, Dublin, CA, United States) by using the 24-2 SITA standard strategy. The deterioration of visual fields in both eyes showed marked central scotoma in right eye than left eye (Supplementary Figures 3A,B).

**FIGURE 4:**
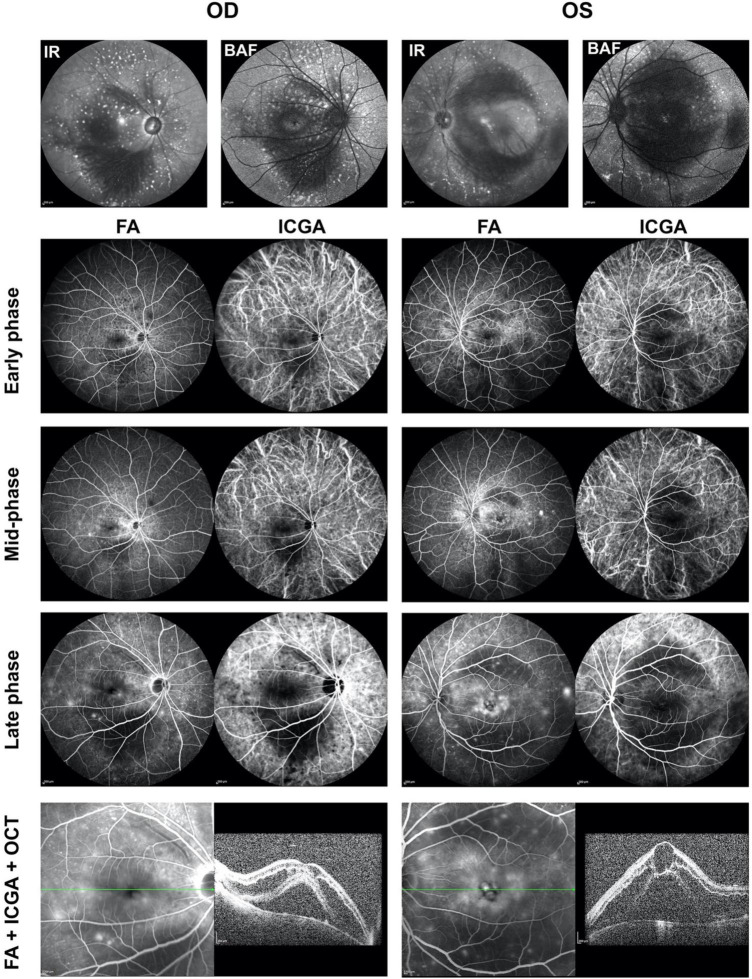
Fluorescein angiography (FA) and indocyanine green angiography (ICGA) showed bilateral changes of retinal and choroidal vessels. Infrared (IR) image showed patched hypo-reflection in macula and hyper-reflective flecks surrounding the macula and optic discs in both eyes. Background autofluorescence (BAF) image showed patched hypo-autofluorescence in macula and hyper-autofluorescence flecks surrounding the macula and optic discs in both eyes. Both FA and ICGA showed delayed and slow perfusion of the retinal and choroidal vessels. The flecks were early blocking with late staining. FA showed macular leakage with pronounced cystoid macular edema, deep masking in the area of the choroidal detachment. ICGA showed dilation and congestion of the choroidal vessels, especially vortex veins, in both eyes. SD-OCT with the Heidelberg Spectralis showed cystoid macular edema with intraretinal fluid and subretinal fluid accumulation in both eyes. FA, fluorescein angiography; ICGA, indocyanine green angiography; IR, infrared; BAF, background autofluorescence; OD, right eye; OS, left eye.

When performing fluorescein angiography (FA) and indocyanine green angiography (ICGA) with SPECTRALIS Heidelberg Retina Angiography, the results of both FA and ICGA showed delayed perfusion and slow flow in both retinal and choroidal vessels (Figure 4). FA shows marked leakage of macula and optic nerve head with time, cystoid macular edema, early blocking with late staining of above flecks shown in the backgrounds of infrared (IR) and autofluorescence (BAF) in both eyes; as well as mild leakage in peripheral retina; while ICGA showed dilated and tortuous vortex veins without obvious leakage in early phase and mid-phase (Figure 4). Both FA and ICGA showed the mask of the choroidal detachment (hypo-fluorescence) in both macula. SD-OCT with the SPECTRALIS Heidelberg showed cystoid macular edema with intraretinal fluid and subretinal fluid accumulation in both eyes (Figure 4). In the mid-phase of FA and ICGA, the images of four quadrants including superior, nasal, inferior, and temporal were taken (Supplementary Figure 4). FA results showed that the peripheral retina vessels, especially the temporal to the fovea, were dilated with leakage over time; and ICGA demonstrated the dilation, tortuosity and congestion of choroidal vessels, which was more obvious for the vortex veins (Supplementary Figure 4).

Based on the patient history and these findings, we made the tentative diagnosis of venous stasis retinopathy and choroidopathy with CSCR-like abnormalities as a result of PAH undertreated with long-term of sildenafil.

## Outcome and follow-up

Close follow-up of PAH and ocular complications was advised. The patient was under an intense treatment of PAH, including sildenafil (2.5 mg, bid), ambrisentan (5 mg, qd), digoxin (0.125 mg, qd), torasemide (10 mg, qd), spironolactone (20 mg, bid), and potassium chloride (0.5 g, tid). For both eyes, brinzolamide eye drops (S.A.Alcon-Couvreur N.V.) were used three times per day. About 6 weeks later, the patient came back for ocular examination. The patient reported the improved visual acuity and decreased metamorphopsia. The BCVA (logMAR) was FC/10cm in right eye and 0.52 in left eye. The mean refractive error was –2.00 diopter (right eye) and –0.75 diopter (left eye). The intraocular pressure was 11.4 mmHg (right eye) and 10.3 mmHg (left eye).

The OCTA b-scan demonstrated significant reduction of the macular edema in both eyes (Supplementary Figures 5–D). OCTA b-scan showed that disruption of ellipsoid zone and deposit of hyperreflective material in subfoveal field in right eye (Supplementary Figure 5A); while the disruptions of Henle fiber, external limiting membrane and ellipsoid zone were evidenced in left eye, along with the cystoid edema and the deposit of hyperreflective material in subfoveal field (Supplementary Figure 5C). The vessel densities in SCP were lower in right eye than that in left eye in each sections of ETDRS-based grid (Supplementary Figures 5A,C). The direct comparisons of OCTA between the first visit and follow-up clearly demonstrated the significant decrease of central macular thickness and absorption of both intraretinal and subretinal fluid in both right eye (Supplementary Figure 5B) and left eye (Supplementary Figure 5D). The visual field examination with perimetry showed the aggregation of visual field loss in right eye, while improvement in left eye (Supplementary Figures 3C,D).

## Discussion

Pulmonary arterial hypertension, if left untreated or undertreated, may result in pulmonary vascular resistance, right heart failure, and death. Ocular complications related to PAH occur as a result of elevated venous pressure in the superior vena cava and in the ophthalmic veins, which cause dilation of the ocular veins, resulting in congestion of the choroid and leading to complications such as ciliary detachment, central retinal vein occlusion, acute serous retinal detachment, macular edema, retinal neovascularization, choroidal effusions, chemosis, angle-closure glaucoma, transient myopia, and etc. (10). Martiano et al. reported a case of a 44-year-old woman with idiopathic PAH, who presented as acute transient visual loss, serous macular detachment and retinal folds (11).

In this study, we reported a case of bilateral CSCR-like abnormalities in a undertreated PAH patient (Figures 2,3). The prominent dilation and tortuosity of episcleral and conjunctival vessels were evidenced in both eyes. The fundus demonstrated chronic CSCR-like signs, including macular edema (intraretinal fluid and subretinal fluid), but the characteristic leakage of CSCR, e.g., visible pinpoint focal leakage, was lack in FA and ICGA examinations.

Although sildenafil was demonstrated safe in the eye when systemic use (9), the side-effects related to sildenafil in the eye were also reported, including phototransduction impairment of photoreceptors, visual disturbance, non-arteritic anterior ischemic optic neuropathy, central retinal vein occlusion, optic atrophy, serous macular detachment, CSCR as well as the increase of choroidal blood flow velocity, etc. (6, 8, 12–16). The ocular complications occur mainly due to the enhanced pressure in the superior vena cava and in the ophthalmic veins, resulting in dilation of the ocular veins and congestion of the episcleral and conjunctival vessels as well as the choroidal vessels. In this case, the patient was treated with low-dose sildenafil (2.5 mg, bid), which was significantly lower than the recommended dosage (20–80 mg, tid) to treat PAH (3). Thus the direct causal effect of low-dose sildenafil on CSCR-like abnormalities can be largely excluded.

In this patient, the obvious dilation and tortuosity of episcleral and conjunctival vessels, as well as the retinal and choroidal vessels (Figures 1, 4 and Supplementary Figure 4) were evidenced due to PAH. Both intraretinal and subretinal fluid on OCTA b-scan (Figure 3) might be due to the breakdown of blood-retinal barrier (BRB), leading to fluid accumulation in retinal parenchyma and subretinal space. Previous study showed that non-arteritic anterior ischemic optic neuropathy, although rare, was reported in patients with sildenafil treatment (16). In the present study, optic atrophy was evidence in right eye with normal intraocular pressure. Due to no history documentation, it is hard to know whether this optic atrophy was related to non-arteritic anterior ischemic optic neuropathy (Figure 2 and Supplementary Figure 2).

Although the exact mechanism for serous retinal detachment and intraretinal fluid associated with PAH remains unknown, an increase in choroidal dilation and congestion was found with FA and ICGA. The above changes were resolved and improved 6 weeks after the first visit. Treatment of ocular complications associated with PAH mainly involves optimal control of PAH and ophthalmic supportive treatment to prevent severe ocular complications. Considering the prescribed medicine, the reduction of the macular edema might be to the combinational effect of digoxin, torasemide, and spironolactone, although the spontaneous recovery cannot be excluded. Digoxin is used for heart failure. Torasemide is a potent loop diuretic with potential to treat congestive heart failure (17). Spironolactone, as the first steroidal competitive mineralocorticoid receptor antagonist drug, was approved for the treatment of hypertension. It was also demonstrated with efficacy in the treatment of chronic CSCR (18). The combinational effect of these medications well controlled PAH, which might help the patient for the rapid resolution of both the intraretinal and subretinal fluid.

The limitation of this study included no history documentation of the previous eye conditions, lack of gene mutation screening for PAH, lack of family history record of eye and systemic conditions, the direct causal-effect of sildenafil on CSCR, and etc. Besides, the present study was lack of the changes of the choroidal thickness as well as the vascularity index of the choroid in this patients at baseline and during follow-up. In this study, we mainly focused on the changes of the retina, such as the central macular thickness and the vessel densities at baseline (Figure 3) and follow-up (Supplementary Figure 5). The changes of above parameters in choroid might provide new prognostic biomarkers for patients affected by such disease, which deserved further exploration.

In conclusion, we reported a bilateral CSCR-like abnormalities and congestion of ocular vessels in a undertreated PAH patient. Thus, the PAH patients should be educated on the risk of potential visual adverse effects if undertreatment.

## Conclusion

Patients with PAH should be closely followed-up and intensively treated. These patients should be educated on the risk of potential visual adverse effects if undertreatment. Undertreated PAH may complicate the congestion of the choroid and induce CSCR-like abnormalities.

## Data availability statement

The original contributions presented in this study are included in the article/Supplementary material, further inquiries can be directed to the corresponding author.

## Ethics statement

This study is a retrospective cohort study (ClinicalTrials.gov number, https://www.chictr.org.cn, ChiCTR2000038911), and complies with the tenets of the Declaration of Helsinki. The study approved by the Clinical Research Ethical Committee of Shanghai General Hospital affiliated to Shanghai Jiao Tong University, Shanghai, China (Permits No. 2020KY205-2). The patients/participants provided their written informed consent to participate in this study. Written informed consent was obtained from the individual(s) for the publication of any potentially identifiable images or data included in this article.

## Author contributions

JFZ and XRZ were ophthalmologists, reviewed the literature, and contributed to manuscript drafting. JXZ and LMG reviewed the literature and contributed to manuscript drafting and discussion. XRZ, JXZ, and HZ analyzed and interpreted the imaging findings. JFZ and LMG contributed funding acquisition. JFZ was guarantor of this work, who had full access to all the data in this study and took responsibility for the integrity and accuracy of the data. All authors issued final approval for the version to be submitted.
