# Supplementary material for: Case report: Bilateral central serous chorioretinopathy-like abnormalities in a man with pulmonary arterial hypertension
Source: Front Med (Lausanne). 2022 Aug 1;9:983548. doi: 10.3389/fmed.2022.983548 (PMC9376321; doi:10.3389/fmed.2022.983548)
Supplement: Supplementary file 1 [file Data_Sheet_1.pdf]

SUPPLEMENTARY FIGURES

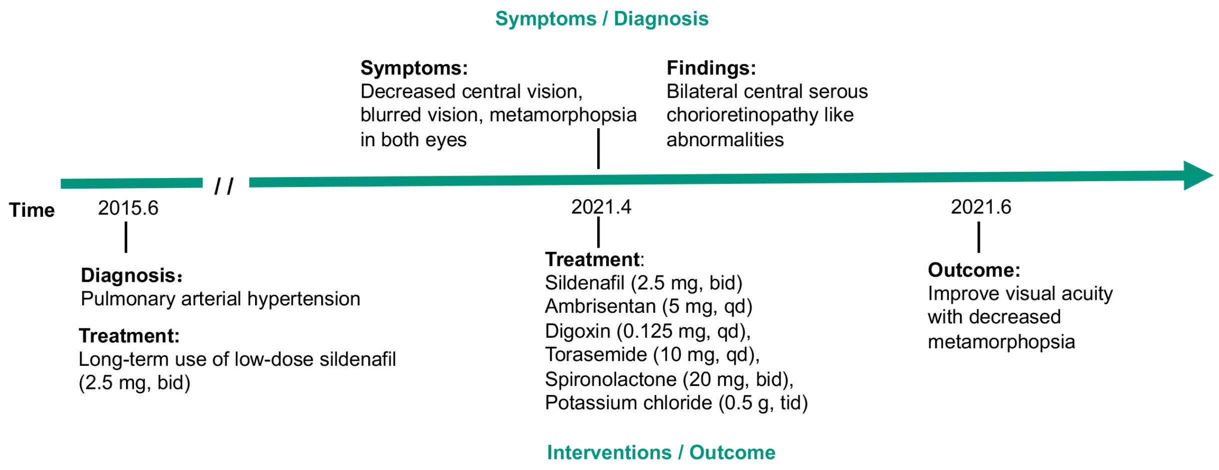

Supplementary Figure 1 Clinical timeline of a 45-year-old male diagnosed of PAH and treated with long-term low-dose sildenafil. This case showed long-term undertreated PAH might cause the congestion of the choroid and induce CSCR-like abnormalities.

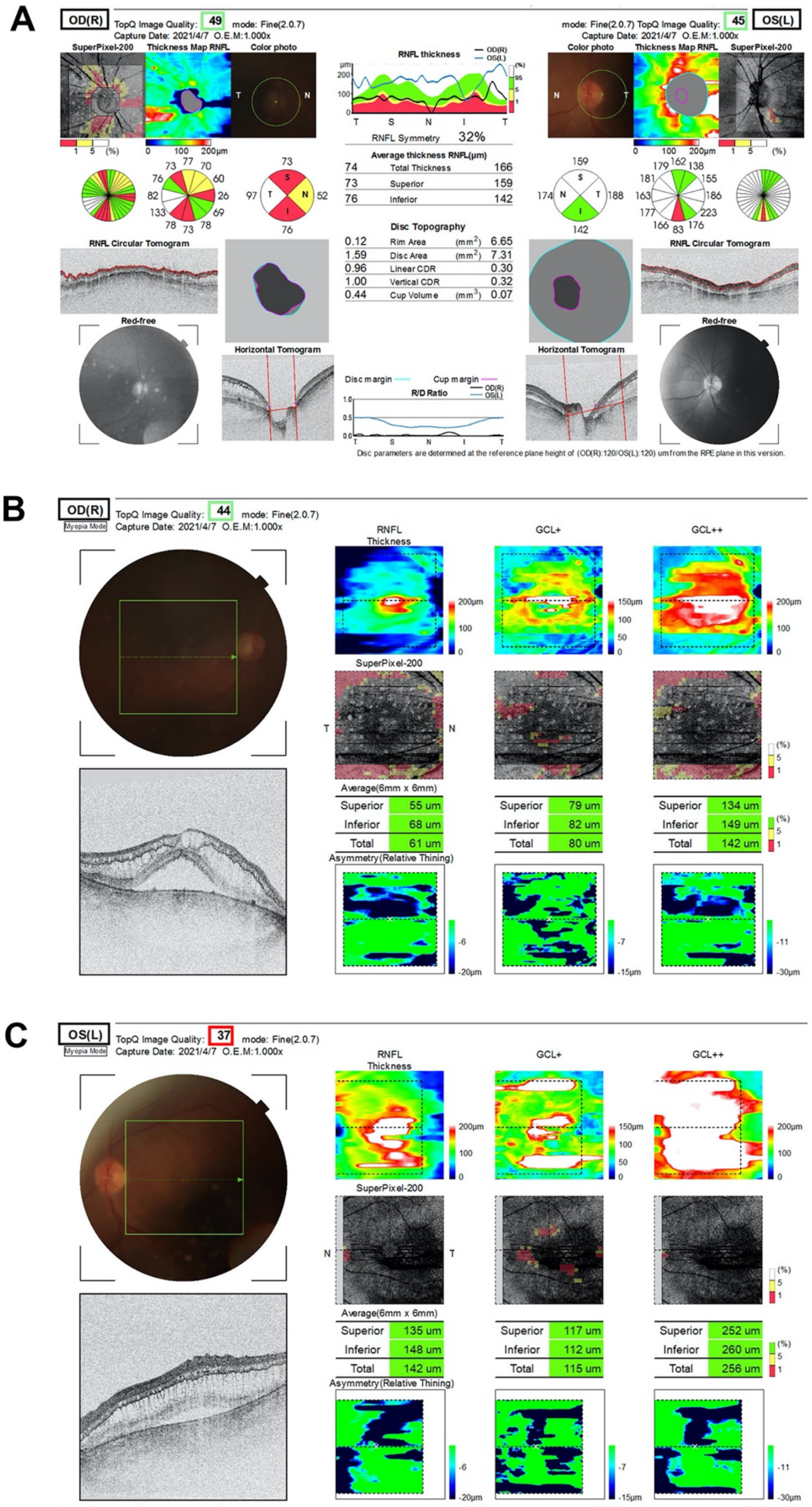

Supplementary Figure 2 The thickness analysis of both optic disc and macula by swept-source optical coherence tomography (SS-OCT). (A) The thickness of retinal nerve fiber layer (RNFL)

of optic disc and disc topography in both eyes on SS-OCT. The thickness of RNFL, GCL+ and GCL++ in right eye (B) and left eye (C). RNFL, retinal nerve fiber layer; GCL+, the thickness of RNFL and ganglion cell layer; GCL++, the thickness of RNFL, ganglion cell layer and inner plexiform layer.

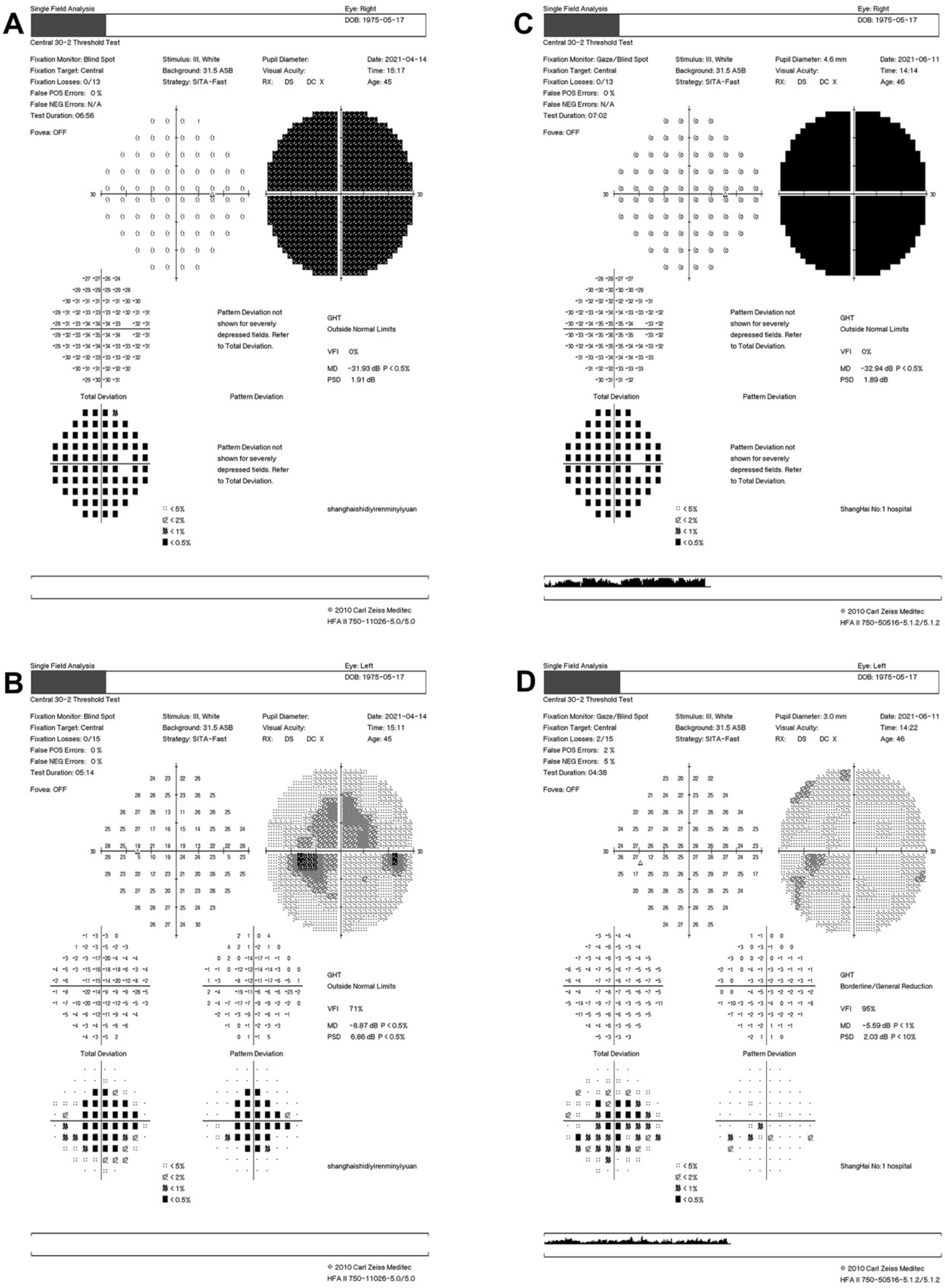

Supplementary Figure 3 Visual field examination of both eyes at first visit (A&B) and follow-up (C&D). Standard automatic perimetry examination shows the significantly decreased visual field with marked central scotoma in right eye (OD) (A) and relatively less central scotoma in left eye

(OS) (B). At follow-up, there was a little deterioration for OD (C), while mild improvement with decreased central scotoma in OS (D).

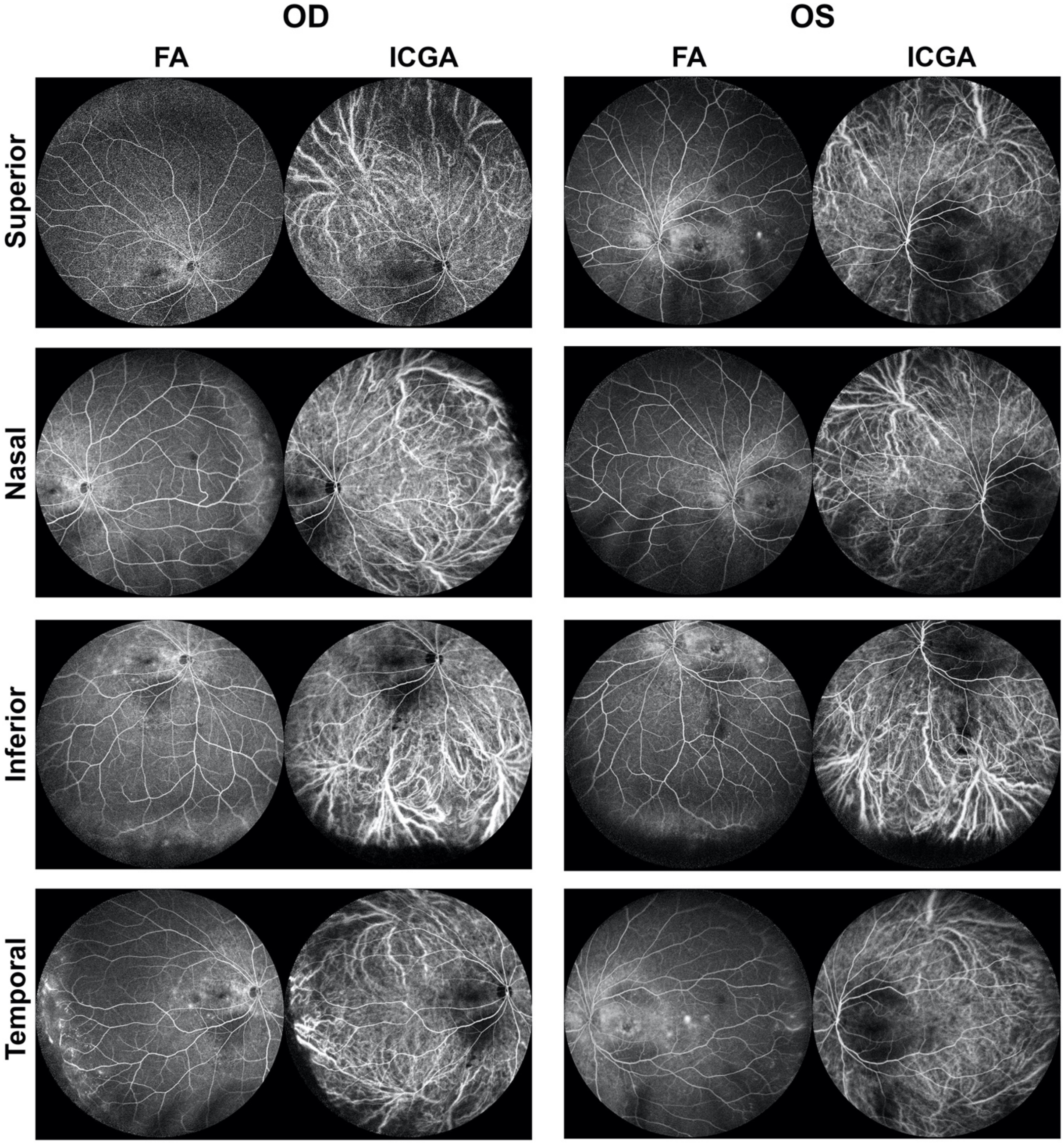

Supplementary Figure 4 The four quadrants of the fundus in both fluorescein angiography (FA) and indocyanine green angiography (ICGA). Marked dilation of temporal retinal vessels with leakage were observed with FA. The prominent dilation, tortuosity and congestion of choroidal vessels and vortex veins were noticed with ICGA. FA, fluorescein angiography; ICGA, indocyanine green angiography; OD, right eye; OS, left eye.

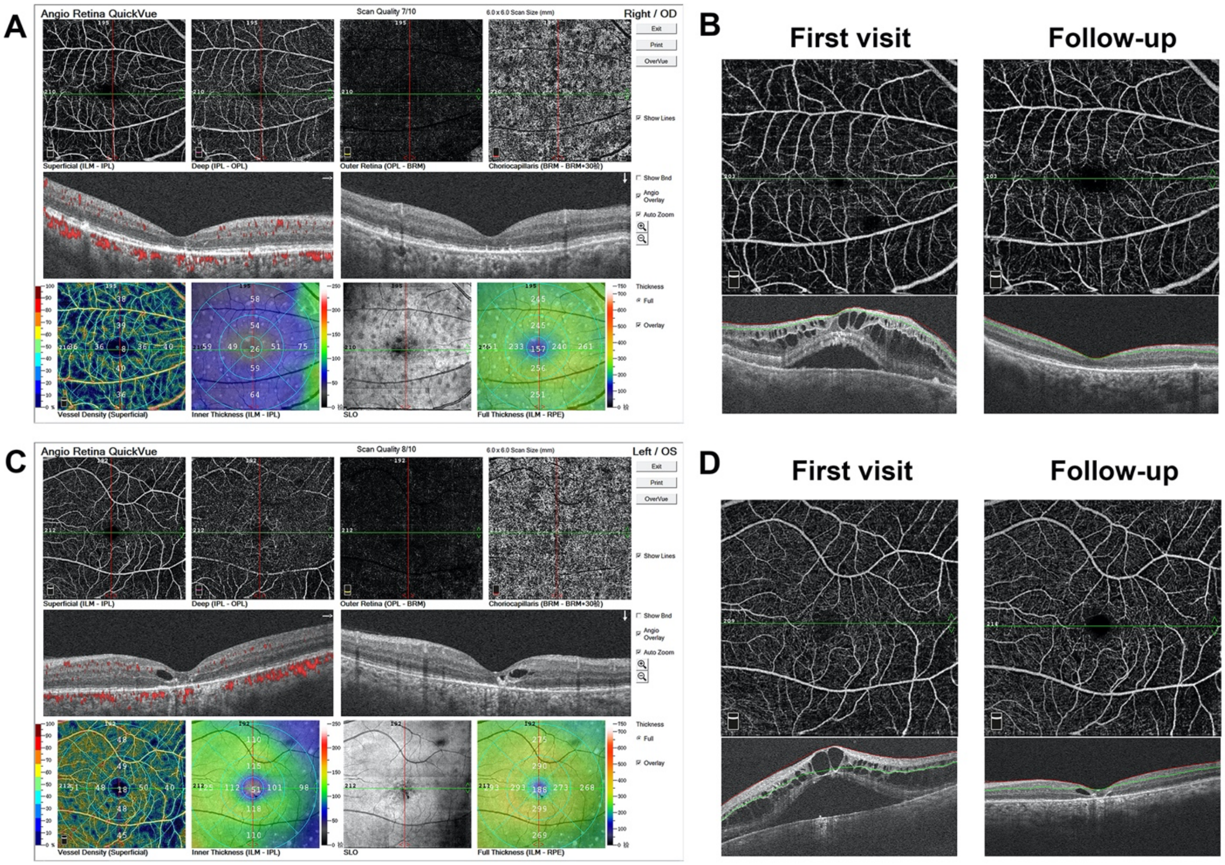

Supplementary Figure 5 Optical coherence tomography angiography (OCTA) examination of both eyes during follow-up. The macular edema were greatly decreased and the fluid was significantly resolved in right eye (A-B) and left eye (C-D). The comparison of the changes of macula between the first visit and follow-up demonstrated the significant decrease of macular edema in right eye (B) and left eye (D).
